# MR‐based CT metal artifact reduction for head‐and‐neck photon, electron, and proton radiotherapy

**DOI:** 10.1002/mp.13729

**Published:** 2019-08-10

**Authors:** Jonathan Scharff Nielsen, Koen Van Leemput, Jens Morgenthaler Edmund

**Affiliations:** ^1^ Department of Health Technology Technical University of Denmark 2800 Kgs. Lyngby Denmark; ^2^ Radiotherapy Research Unit, Department of Oncology, Gentofte and Herlev Hospital University of Copenhagen 2730 Herlev Denmark; ^3^ Department of Radiology Massachusetts General Hospital, Harvard Medical School Boston MA 02114 USA; ^4^ Niels Bohr Institute University of Copenhagen 2100 Copenhagen Denmark

**Keywords:** Bayesian modelling, computed tomography, CT metal artifact reduction, proton therapy, radiotherapy

## Abstract

**Purpose:**

We investigated the impact on computed tomography (CT) image quality and photon, electron, and proton head‐and‐neck (H&N) radiotherapy (RT) dose calculations of three CT metal artifact reduction (MAR) approaches: A CT‐based algorithm (oMAR Philips Healthcare), manual water override, and our recently presented, Magnetic Resonance (MR)‐based kerMAR algorithm. We considered the following three hypotheses: I: Manual water override improves MAR over the CT‐ and MR‐based alternatives; II: The automatic algorithms (oMAR and kerMAR) improve MAR over the uncorrected CT; III: kerMAR improves MAR over oMAR.

**Methods:**

We included a veal shank phantom with/without six metal inserts and nine H&N RT patients with dental implants. We quantified the MAR capabilities by the reduction of outliers in the CT value distribution in regions of interest, and the change in particle range and photon depth at maximum dose.

**Results:**

Water override provided apparent image improvements in the soft tissue region but insignificantly or negatively influenced the dose calculations. We however found significant improvements in image quality and particle range impact, compared to the uncorrected CT, when using oMAR and kerMAR. kerMAR in turn provided superior improvements in terms of high intensity streak suppression compared to oMAR, again with associated impacts on the particle range estimates**.**

**Conclusion:**

We found no benefits of the water override compared to the rest, and tentatively reject hypothesis I. We however found improvements in the automatic algorithms, and thus support for hypothesis II, and found the MR‐based kerMAR to improve upon oMAR, supporting hypothesis III.

## 1 Introduction and Purpose

Metal implants in patients scanned with x‐ray computed tomography (CT) lead to potentially severe cupping and streak artifacts, as the model relating the reconstructed attenuation coefficients to the x‐ray measurements breaks down. Such image corruption may be critical to the accuracy of the electron density and relative stopping power (RSP) estimates needed for external beam radiotherapy (RT) dose calculations. Errors in these estimates directly translate to errors in, for example, the calculated water equivalent thickness (WET) and particle range. The CT additionally provides image material for organ and lesion delineation, whose accuracy could be reduced by the artifacts.^1^, ^2^, ^3^

With photon radiation, the dose plan errors from the artifacts may be small, since photon RT plans generally use arcs or multiple beam angles and may accordingly be relatively robust to errors in the dose deposited by small subsets of the beams.^1^, ^3^, ^4^ Photon absorption is in addition relatively insensitive to tissue variations. For particles, on the other hand, here electrons and protons, the RSP is highly sensitive to tissue variations and the plans typically contain only 1–3 beams.^1^, ^3^, ^4^, ^5^ The resulting dose uncertainties in electron and (the increasingly used) proton therapy can be a major concern for head‐and‐neck (H&N) RT patients with tumors simultaneously close to critical organs at risk (OARs) and the frequently corrupted oral region; it also decreases the degrees of freedom during dose planning.^5^, ^6^

To potentially reduce dose calculation uncertainties, metal artifacts may be manually replaced with bulk CT values (typically water) by an experienced dosimetrist or similar, but this is time‐consuming and subject to systematic bias and human error.^7^, ^8^ Alternatively, automatic metal artifact reduction (MAR) algorithms may be employed, typically supplied by the vendor of the CT scanner as a commercial add‐on. Such vendor solutions must be scrutinized before clinical use to gauge their efficacy and possible limitations.

An example is the clinically used oMAR algorithm^9^ (MAR for orthopedic implants, Philips Healthcare) that provides visual improvements, but has been found to leave behind residual streaks close to the implants.^6^, ^10^, ^11^ These lead to associated RSP estimation errors and thus imperfect WET estimates for proton RT,^6^ as well as findings of minimal photon dose improvement in the oral cavity (with a closed mouth).^1^, ^11^ Consequently, the residual metal artifacts cannot necessarily be neglected during RT dose planning causing a potential loss of automation and accuracy.

Examples of more accurate MAR algorithms may be found among the numerous, well‐documented MAR alternatives in the literature. The existing approaches span from fast and simple raw data interpolation/replacement schemes and image space methods^12^, ^13^, ^14^, ^15^ to complex and slow full iterative reconstruction algorithms,^16^, ^17^ and offer alternatives suitable for a wide range of diverse situations and levels of complex implementation.

We recently presented a novel example of one such alternative, which, in addition to the corrupted CT information, incorporates complementary image information from magnetic resonance imaging (MRI) using kernel regression (kerMAR).^18^ Taking advantage of the less artifact‐corrupted MRI that can be acquired for H&N RT to aid in tumor and OAR delineation, kerMAR uses kernel regression on CT value/MR image patch pairs along with a noise model of the CT artifacts to estimate the true CT values underlying the artifacts. This leads to potential improvements, in particular close to the metal implants where purely CT‐based algorithms are the least effective.

The availability of the mentioned MAR options gives rise to some clinically relevant questions, which we will consider by testing the following three hypotheses (H):

HI: Simple manual water override provides significant benefits over all other MAR alternatives. HI questions the effectiveness of the manual method. HII: The automatic MAR algorithms (kerMAR and oMAR) provide significant benefits over the uncorrected filtered back projection CT (FBP). HII questions the effectiveness of the automatic methods. HIII: The MR‐based kerMAR algorithm is superior to the clinically used oMAR algorithm. HIII questions the effectiveness of the novel, MR‐based alternative.

We investigate these three hypotheses using a phantom as well as retrospective H&N patient data, and evaluate the level of artifact corruption via image metrics that quantify the amount of low and high intensity artifacts. We also investigate the impact of the MAR algorithms on photon, electron, and proton maximum depth/particle range estimates in the dose calculations.

## 2 Materials and Methods

### 2.1 The MAR algorithms

This study considers the three MAR algorithms outlined below (a, b, and c respectively). A schematic illustration can be found in the supplementary material (Section 1, Fig. S1) along with further details.

- *kerMAR* 18 is an image space, Bayesian inference algorithm that uses kernel regression on aligned uncorrupted CT values and cuboidal MRI *patches* (vectors of MRI voxel intensities from local spatial contexts) in the patient volume. It estimates a prior distribution of the true CT value *y* given the corresponding MRI patch **m**, *p*(*y*|**m**). Assuming additive Gaussian artifact noise and given an observed **m** centered on a corrupted location as well as the corresponding corrupted CT value *t*, the posterior distribution *p*(*y*|*t,* **m**) is then constructed. Calculating the expectation value of *y* over this distribution yields the final CT value estimate.^18^ Not only relying on CT information, a potential for artifact reduction in highly corrupted regions is possible.
- *oMAR* 9 is an iterative algorithm that combines image processing and projection replacement. It iteratively improves a tissue‐classified image with consequently suppressed artifacts, which is used to simulate the CT x‐ray measurements by forward projection^19^ and thus approximate an artifact‐free acquisition. This approximation is then expected to improve over the iterations.^9^
- *Manual override* 7, 8 techniques address the metal artifacts by visually replacing artifact‐corrupted regions with a bulk CT value. Since the oral cavity is largely water equivalent, a plausible CT value is 0 Hounsfield Units (HU),^18^ leading to a water override. How this override is performed depends on the local clinical practice; in our approach, we replaced obviously corrupted soft tissue regions as well as severely corrupted high intensity regions by this reproducible, generic value.

### 2.2 Study overview and materials

We split the study into two parts: (a) A phantom study where we evaluated the MAR algorithms on a veal shank without (ground truth) and with a set of inserted metal pins; and (b) a retrospective study on nine randomly selected H&N RT patients.

The CT images were acquired using a Philips Brilliance Big Bore scanner, 120 kVp and resolution (0.5 × 0.5 × 2.0) mm^3^. The MRIs were acquired on a Philips Panorama 1.0T HFO scanner using a 2D T1w sequence (*TE/TR* = 10 ms*/*520*.*2 − 572*.*2 ms) at resolution (0.5 × 0.5 × 5.5) mm^3^. For the MR‐based MAR algorithm kerMAR, the MRIs were rigidly co‐registered to the CTs using mutual information 20, 21 and resampled to the CT resolution.

**Figure 1:**
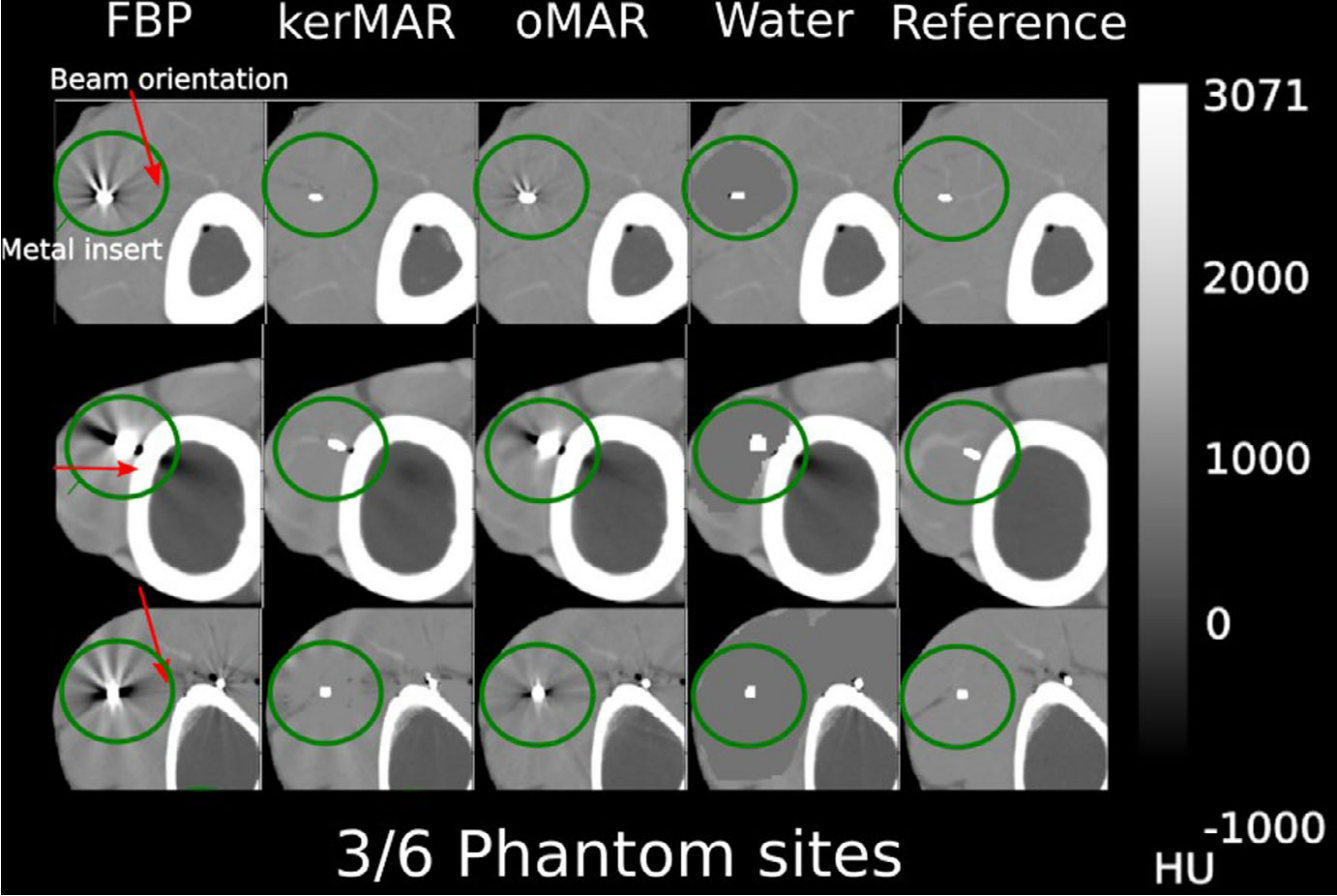
Axial filtered back projections (FBPs), metal artifact reduced images (kernel regression metal artifact reduction (kerMAR); metal artifact reduction for orthopedic implants (oMAR); water override) and uncorrupted reference slices of the veal shank phantom. The images are in the central plane of the therapeutic beams, (orientations shown by red arrows). Results are shown for 3 out of 6 inserted metal pins. [Color figure can be viewed at http://www.wileyonlinelibrary.com/]

**Figure 2:**
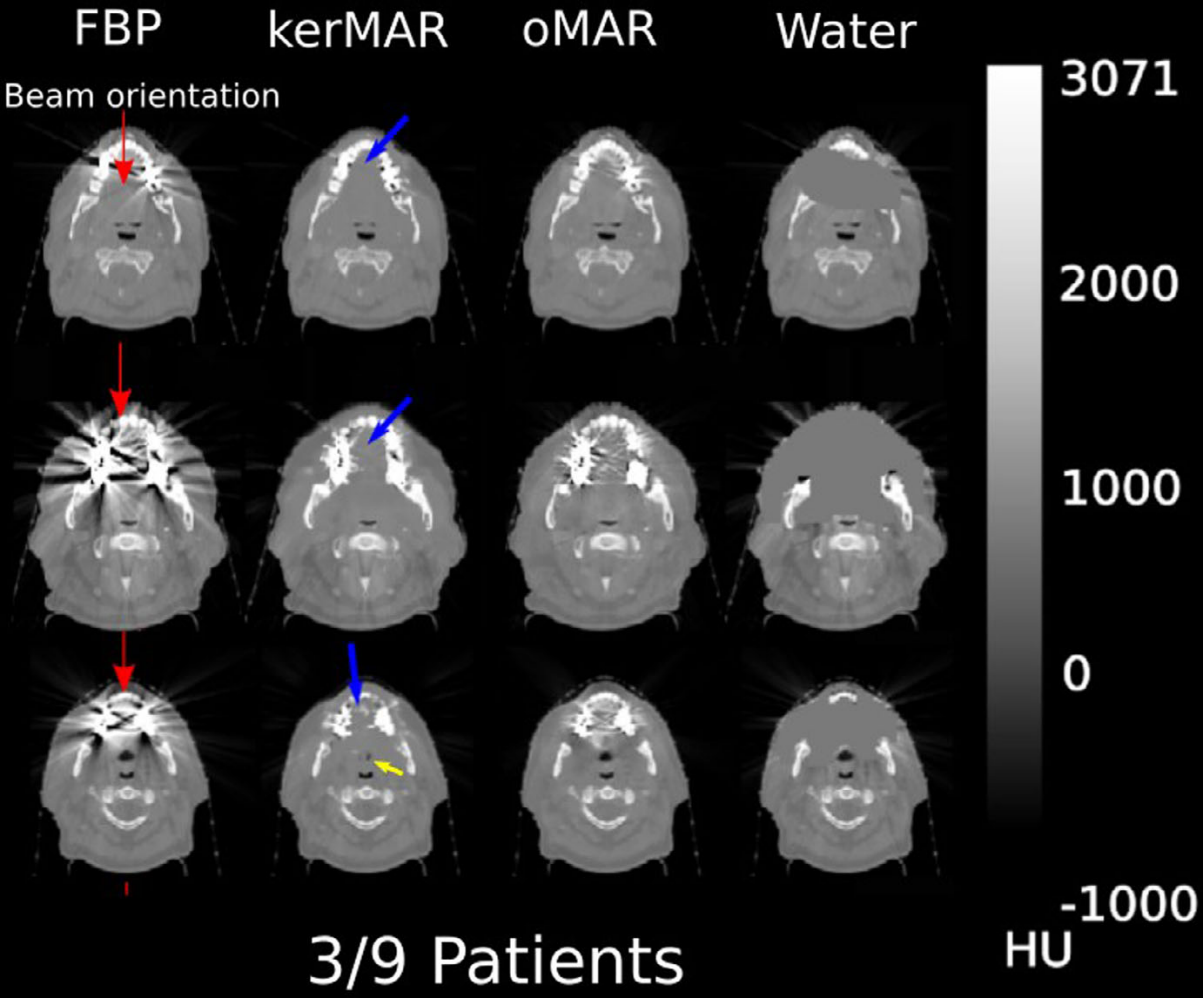
Axial filtered back projection (FBP) and metal artifact reduced slices in 3 of 9 patients. Red arrows as in Fig. 1. Blue arrows indicate potential benefits of kernel regression metal artifact reduction (kerMAR) over metal artifact reduction for orthopedic implants (oMAR). The yellow arrow indicates kerMAR‐introduced artifacts near the trachea caused by different magnetic resonance and computed tomography acquisition positions. [Color figure can be viewed at http://www.wileyonlinelibrary.com/]

For all pins and patients, we acquired the FBP CT and oMAR as well as the T1w MRI, performed water override and calculated the kerMARs. Representative, axial CT image slices for the phantom and patients are shown in Figs. 1 and 2, respectively.

## 3 Experiments

We evaluated the MAR algorithm performance by two metrics: (a) We quantified the amount of artifacts by the number of voxels with unexpectedly low and high CT values (streaks) as compared to an uncorrupted adjacent reference volume; (b) We quantified the impact on the dose distributions by the calculated depth at maximum dose (photons) and the effective range (particles) for beams angled through the corrupted regions (red arrows Figs. 1 and 2).

### 3.1 Image analysis of the artifacts

**Figure 3:**
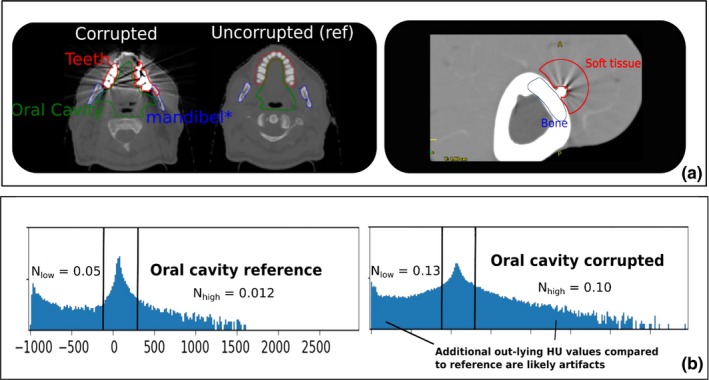
Image analysis of the metal artifacts. (a): For the patients (left), uncorrupted parts of the regions of interest (ROI) were used as reference. For the phantom (right), the reference was the implant‐free computed tomography scan. (b): Histograms of the corrupted and uncorrupted parts of the oral cavity. The corrupted part (right) shows an increase in the fraction of voxels outside the expected HU range (*N_low_* and *N_high_*), indicating artifacts as compared to the reference (left). Similar analysis was made for all phantom/patient ROIs. [Color figure can be viewed at http://www.wileyonlinelibrary.com/]

Figure 3 illustrates the image analysis. For the patients, we acquired the clinical delineations of the oral cavity (including parts of the trachea) and mandible, and manually delineated the teeth. For each patient, we split the regions of interest (ROIs) into a corrupted region and an uncorrupted reference region by visual inspection [Fig. 3(a), left].

We then calculated the following differences in frequency between the corrupted and uncorrupted regions for the low and high tails of the CT value histogram [Fig. 3(b)]:

$$\delta N_{low/high}=\left(N_{low/high^{\mathrm{corr}}}-N_{low/high^{\mathrm{uncorr}}}\right).$$

N_low/high_^corr/uncorr^ are here the ratios of the voxels below and above certain thresholds compared to the total. We defined the thresholds by identifying the lowest and highest attenuating common tissue types in each of the ROIs. For all but the tooth enamel, we estimated the CT value of these tissue types from the composition and mass density data in ICRU 46,^22^ the scanner effective energy (75*.*2 keV) and NIST^23^ mass attenuation coefficients.

For the enamel, which was not in the ICRU database, we estimated the mass density of its main component (hydroxyapatite) as the average over its molecular constituents from its chemical formula.

This produced the following thresholds: oral cavity: Adipose (−200 HU) to average soft tissue (300 HU); mandible: mandibular (1000 HU) to cortical bone (1500 HU); teeth: cortical bone (1500 HU) to enamel (2600 HU).^22^

For the phantom, we delineated artifact‐corrupted soft tissue and bone regions [Fig. 3(a), right]. For the soft tissue, we used the oral cavity thresholds, but for the shank bone (tibia) we had no reliable information on the composition and instead considered the HU distribution in uncorrupted regions, yielding thresholds of 1500 and 1800 HU, respectively. *δN_high/low_* were defined as the difference to the uncorrupted (implant‐free) CT scan.

### 3.2 Depth/range experiments

**Figure 4:**
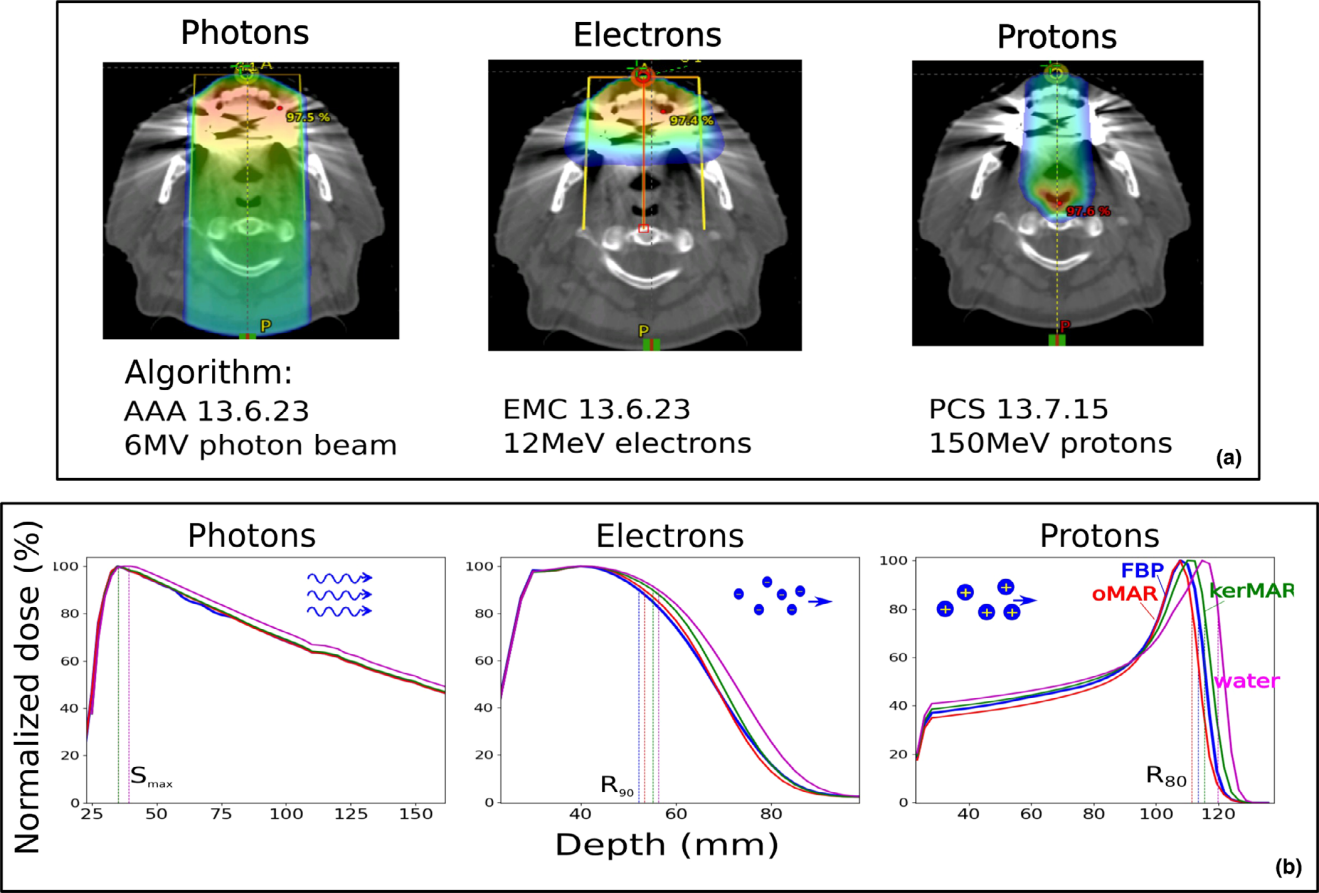
Description of the depth/range estimation setup. (a): Dose color wash from calculations in the treatment planning system Eclipse v. 13.6 (Varian Medical Systems) for photon, electron and proton beams angled through the oral cavity of a H&N patient. (b): Depth‐dose curves along the central profiles of the beams from which the photon depths and particle ranges were derived. The colors correspond to different metal artifact reduction algorithms as indicated in the proton panel (right). [Color figure can be viewed at http://www.wileyonlinelibrary.com/]

For the dose calculation experiments, we investigated the impact of the MAR algorithms on the planned maximum dose^24^, ^25^ (photons) and particle range (electrons and protons). For the electrons, we quantified the particle range by the distal depth at 90% of maximum dose (the *therapeutic range*,^26^ denoted R_90_), and similarly for the protons by R_80_ 27 [see Fig. 4(b)].

For the patients, we intentionally considered the extreme cases where the beams were angled through the corrupted oral cavity [Fig. 4(a)]. For the phantom, we chose beam orientations that were similar to the patient experiments, in that they were near to both artifacts and bone (see Fig. 2). For all beams in phantom and patients, three dose plans were created in Eclipse 13.6 (Varian Medical Systems), using 6 MV photons, 12 MeV electrons, and 150 MeV protons.

From the dose distributions, we extracted the depth and range estimates along the central‐axis depth‐dose curves of each plan. The dose was calculated with a grid resolution of 1 and 2 mm for electrons and protons/photons, respectively. To allow for the detection of variations below this resolution, we cubically interpolated the depth‐dose curves to a resolution of 10^−3^ mm; we investigated the error potential of this strategy in the supplementary material section 3 (see in particular Fig. S3) and found it to be within tolerance.

### 3.3 Statistical analysis

We considered the data from the separate phantom pins and patients as independent observations, and the data from the different MAR algorithms as dependent observations. Our data thus consisted of four repeated measurements (FBP, oMAR, kerMAR, and water override) on N = 6 and N = 9 subjects for the phantom and patients, respectively. We investigated the following three orthogonal contrasts: I: The aggregate (in particular, average) of kerMAR, oMAR, and FBP vs water override; II: FBP vs the aggregate of kerMAR and oMAR; III: oMAR vs kerMAR. All contrasts being orthogonal, the p‐values of the statistical tests described in the next paragraphs were not corrected for multiple comparisons.^28^

For the image analysis, we used a two‐tailed Student's *t*‐test for paired (dependent) observations on the absolute values of the image corruption metrics *δN_high/low_*. We looked for significant differences in these quantities between the contrasted MAR approaches and thus calculated the *t*‐statistic from the mean (∆|*δN_high/low_*|) and standard deviation of the absolute difference between the contrasted terms. A positive mean value implies a smaller |*δN_high/low_*| for the second term (e.g., kerMAR in III) relative to the first term (e.g., oMAR in III), and so a positive test result with a positive mean supports the tested hypothesis, while a negative mean rejects it.

For the phantom depth/range results, we similarly calculated ∆|*δS_max_/δR*_90_ */δR*_80_|, leading to the same interpretation of the results. For the patients, due to the absence of ground truth, we instead calculated the mean absolute difference between the depths/ranges, denoted |∆*S_max_/*∆*R*_90_ */*∆*R*_80_|. This quantity being strictly positive, we performed a one‐tailed Student's *t*‐test of the hypothesis that it was equal to 0.

## 4 Results

### 4.1 Hypothesis I: FBP, oMAR, and kerMAR vs water override

**Figure 5:**
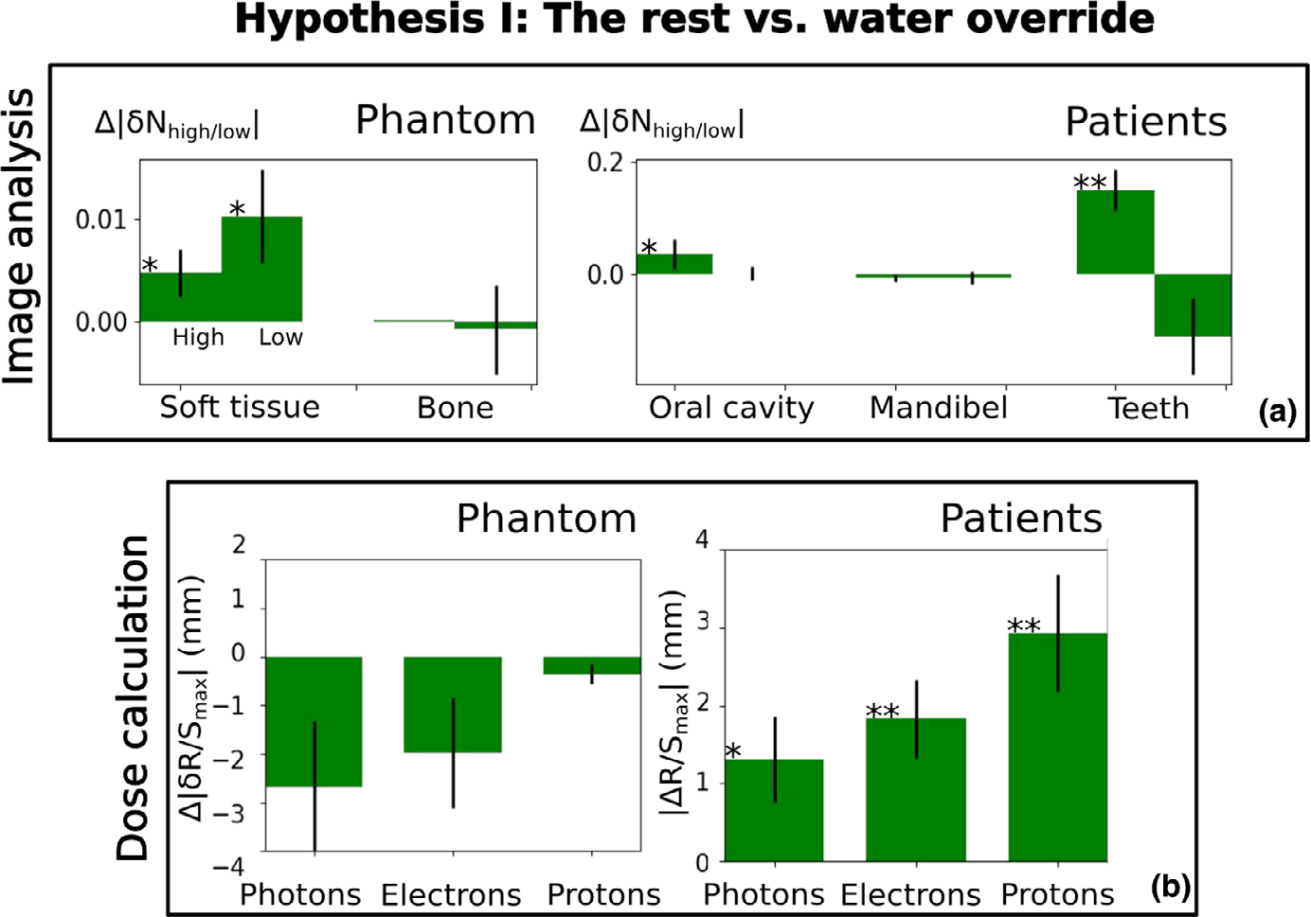
Mean (bar heights) and SDE (error bars) of the variations between the aggregate of kernel regression metal artifact reduction (kerMAR), metal artifact reduction for orthopedic implants (oMAR) and filtered back projection (FBP), and the water override. (a): ∆|*δN_high/low_*| for the phantom (left) and the patients (right). (b): Photon depths and particle ranges, ∆|*δS_max_/δR*_90_ */δR*_80_| for the phantom (left) and ∆|*S_max_/R*_90_ */R*_80_| for the patients (right). Asterisks indicate significance as *P < 0*.*05, **P < 0*.*01. Except for the patient depth and range variations (b, right), positive variations support the hypothesis while negative reject it. [Color figure can be viewed at http://www.wileyonlinelibrary.com/]

Figure 5 shows the aggregate of kerMAR, oMAR, and FBP contrasted with the manual water override. Figure 5(a) shows significantly positive phantom differences in the soft tissue for *δN_high/low_*. In the patients, positive significant differences are observed in *δN_high_* for the oral cavity and teeth, with almost significantly negative ∆*δN_low_* for the teeth. The depth/range results in Fig. 5(b) are nonsignificantly negative for the phantom while the patient results are significant for photons and highly significant for electrons/protons at ∆*R*_90_ = 1*.*9 ± 0*.*3 and ∆*R*_80_ = 3*.*0 ± 0*.*4 mm, respectively; photons show a smaller, less significant variation at ∆*S_max_* = 1*.*2 ± 0*.*4 mm.

### 4.2 Hypothesis II: FBP vs oMAR and kerMAR

**Figure 6:**
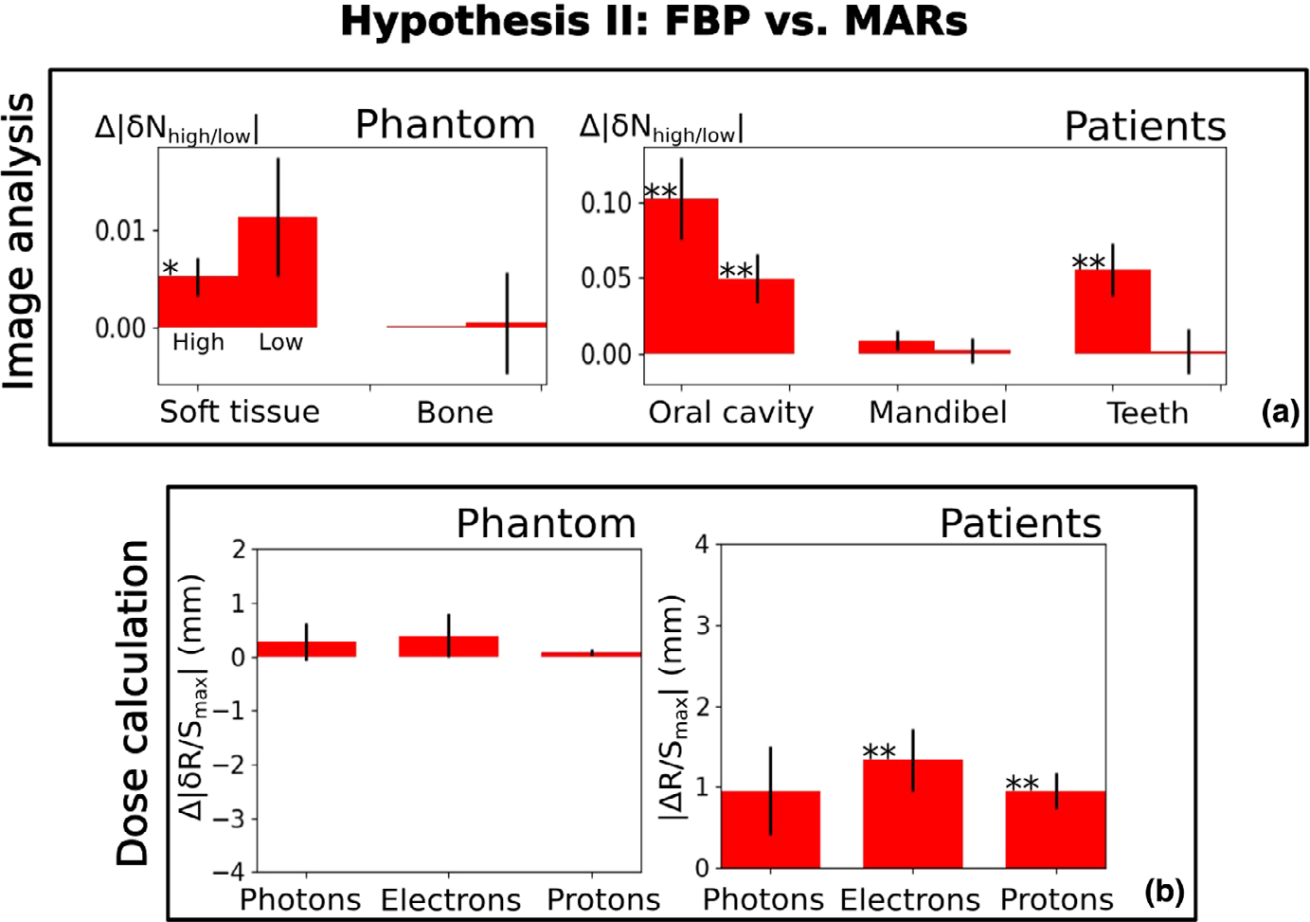
Mean (bar heights) and SDE (error bars) of the variations between the uncorrected filtered back projection (FBP), and the aggregate of metal artifact reduction for orthopedic implants (oMAR) and kernel regression metal artifact reduction (kerMAR). Layout details are identical to fig. 5. [Color figure can be viewed at http://www.wileyonlinelibrary.com/]

Figure 6 displays the contrast between the uncorrected FBP and the aggregate of the automatic algorithms. Figure 6(a) shows only positive values of ∆|*δN_high/low_*|, consistently implying positive benefits of the MARs. We observe positive significance for the phantom soft tissue and patient oral cavity and teeth, but not the phantom bone and the mandible.

In Fig. 6(b), we see no significant phantom dose results; for the patients, however, we observe a highly significant difference with particles of ∆*R*_90_ = 1*.*5 ± 0*.*4 and ∆*R*_80_ = 1*.*0 ± 0*.*3 mm, respectively, but not for photons (∆*S_max_* = 1*.*0 ± 0*.*5 mm).

### 4.3 Hypothesis III: oMAR vs kerMAR

**Figure 7:**
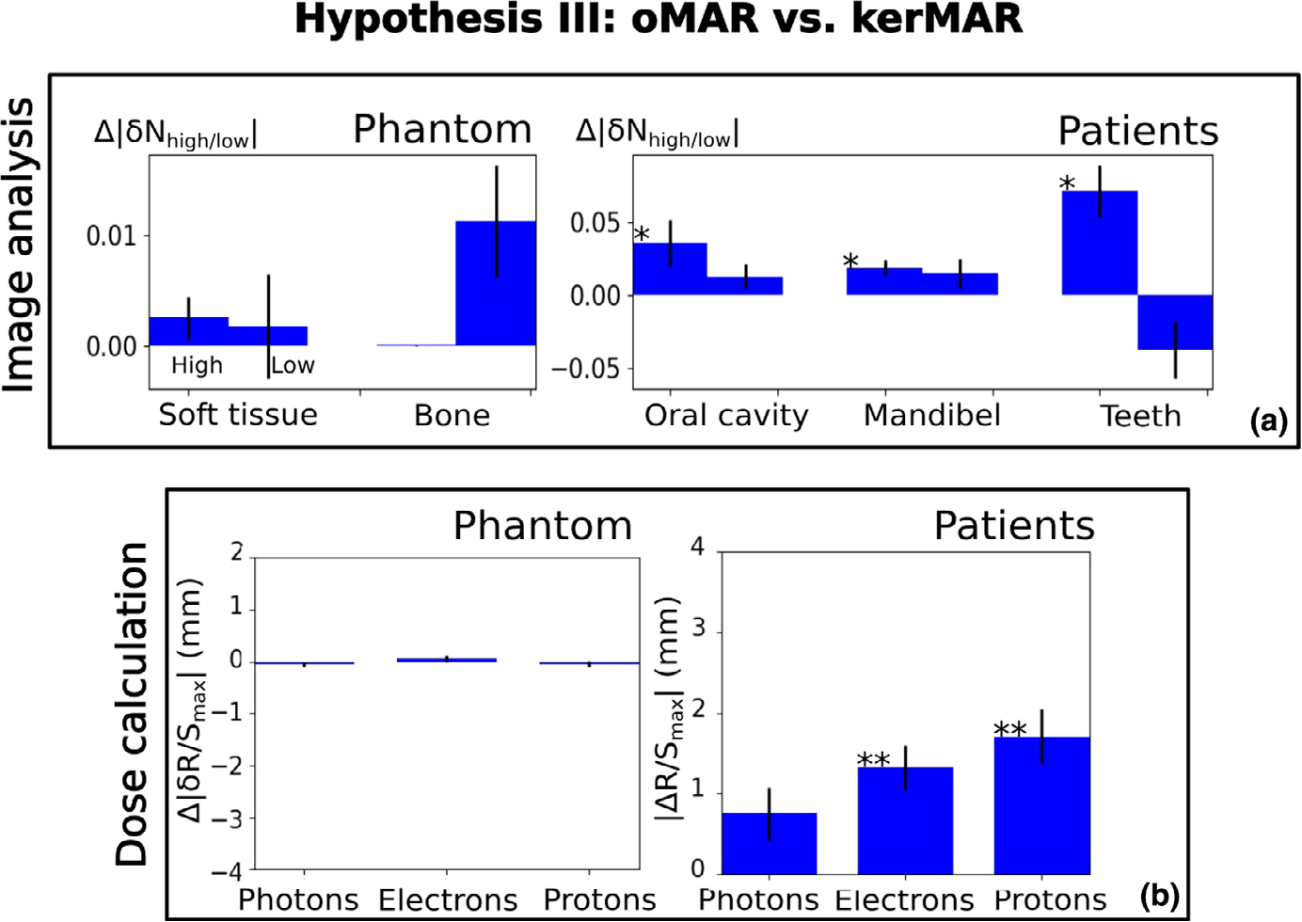
Mean (bar heights) and SDE (error bars) of the variations between metal artifact reduction for orthopedic implants (oMAR) and kernel regression metal artifact reduction (kerMAR). Layout details are identical to fig. 5. [Color figure can be viewed at http://www.wileyonlinelibrary.com/]

Figure 7 contrasts the automatic algorithms oMAR and kerMAR. The image analysis results [Fig. 7(a)] in the phantom are insignificant, while the patient ∆|*δN_high_*| are universally positive and significant. For the depth/range results [Fig. 7(b)], we see insignificant results in the phantom. For the patients, however, we see significant differences with particles of respectively ∆*R*_90_ = 1*.*3 ± 0*.*3 and ∆*R*_80_ = 1*.*8 ± 0*.*4 mm*,* but an insignificant difference for photons (∆*S_max_* = 0.7 ± 0*.*5 mm).

## 5 Discussion

We have considered four MAR approaches in the context of H&N radiotherapy, using phantom and retrospective patient data to evaluate the MAR performance via image quality metrics and the impact on photon, proton and electron maximum depth and range estimates. We focused on three hypotheses of potential clinical relevance.

### 5.1 Hypothesis I (oMAR, kerMAR, and FBP vs water override)

Our first hypothesis was that manual water override would provide a benefit over the alternatives, that is, the automatic MARs (oMAR and kerMAR) and the uncorrected FBP.

In our study, water override showed significant apparent image improvements in the soft tissue and teeth *δN_high_*. These improvements, however, did not lead to an increase in dose accuracy in the phantom, but rather the opposite. This can be explained by the systematic difference between water and the surrounding tissue (40–100 HU), which may also translate to the H&N cases due to water override of the muscular tongue; our phantom study thus suggests caution when performing water override as it may introduce more systematic errors than it removes.

A way to circumvent such errors is to use a more appropriate override HU value of, for example, 60 HU, which leads to more positive results for the phantom in our study (see Fig. S2 in section 2 of the supplement). This strategy may, however, be infeasible in cases with no obvious correct replacement value, such as the entire oral cavity.

For the patients, we observed significant mean depth/range deviations between the water override and the rest (1–3 mm for photons, electrons and protons). This may in part be due to the mentioned systematic errors including override of teeth and air cavities. Our study thus suggests that override of different structures by a single HU value can introduce errors on the order of 3 mm for (worst case) proton dose planning. These are of the same order of magnitude as the errors from metal artifacts reported in the literature.^1^, ^2^, ^3^, ^4^, ^5^, ^29^

A potential limitation to this part of the study is that our chosen override approach, which sometimes replaced high‐intensity regions, for example, teeth, does not reflect the maximum potential of the water override. Our results should rather be viewed as a worst‐case impact of the method.

### 5.2 Hypothesis II (FBP vs oMAR and kerMAR)

Hypothesis II investigated whether the automatic MAR algorithms would improve over an uncorrected FBP image. In terms of image quality, the literature suggests improvements in oMAR in the CT value mean and variance of ~25% in soft tissue, and an improved average in bone areas.^9^, ^10^, ^11^ For protons, these improvements have further been accompanied by proton range estimate improvements of several mm,^3^, ^4^ and WET estimate improvements by a similar amount.^6^ In comparison, photon dose accuracy improvements have been found to be modest, with negligible improvements for beams passing through the oral cavity for patients with closed mouths.^1^, ^11^

In agreement with the literature findings, our H&N patient study showed apparent image quality improvements in a similar magnitude (~5%–10% in STD), which were accompanied by significant 1–1.5 mm particle range and insignificant photon impacts.

### 5.3 Hypothesis III (oMAR vs kerMAR)

The final hypothesis tested whether kerMAR would provide benefits over the clinically used oMAR algorithm. The literature has found oMAR to leave behind residual streaks in highly corrupted regions close to the metal implants. Since the MR‐based kerMAR includes an external source of prior information, it may better handle these residual streaks and thus lead to improvements in both image quality and dosimetric accuracy.^1^, ^6^, ^11^

In our study, kerMAR and oMAR performed similarly for the phantom, both in terms of image quality and dosimetric agreement with the ground truth. The H&N patients, however, showed significant improvements in kerMAR in terms of the image corruption metric *δN_high_*, consistent over the ROIs. From visual inspection (blue arrows in Fig. 2), the source of this improvement appears to be an increased reduction in the residual streaks in the oral cavity.^6^, ^11^

Considering the depth/range results for the patients, while the photon *S_max_* was not significantly impacted by the image improvements, kerMAR did lead to (highly) significant absolute particle range differences from oMAR of ~1 and 2 mm for electrons and protons, respectively. These findings are consistent with a maximal found WET error of ∼4 mm in a hip implant phantom study.^6^

### 5.4 Concerns of significance, application limitation, and clinical implementation

Our reported variations are of several mms, which is near the resolution of both the CT images and dose distributions (0.5–2 mm). This may question their clinical significance for the following reasons: (a) they may appear minor compared to other errors in the clinical workflow, such as delineation, motion, and MRI‐to‐CT co‐registration errors, each of which may be associated with average systematic errors of up to 3 mm^29^; (b) they may have negligible impact on the actual dose distribution. However, it should be noted that our results are average quantities over cases with varying artifact corruption, for which the proton range differences in some cases exceeded 3 mm in all the investigated contrasts. Additionally, the artifacts generally lead to more unpredictable alterations in the dose distribution than a simple translation (see e.g., the proton depth‐dose‐curves in Fig. S3 of the supplement), which may amplify their clinical impact flow, such as delineation, motion, and MRI‐to‐CT co‐registration errors, each of which may be associated with average systematic errors of up to 3 mm.^29^

While the oMAR algorithm is a well‐established commercial solution in clinical use, the MR‐based kerMAR algorithm is a novel alternative that has not been part of a standard clinical workflow. Its design, however, can be well integrated in future clinical implementation: any required steps in the algorithm that are not already part of the clinical workflow are fully automatized, and it requires only a conventional sequence MRI (e.g., T1w) from the patient in consideration, automatically accounting for potential sequence variations. It is also reasonably fast, currently requiring 10–30 min on a laptop with potential for significant optimization.

kerMAR, however, has certain potential drawbacks that one should bear in mind.^18^ In particular, when the MRI and CT are not well aligned, the kerMAR may introduce artifacts as the MR‐based prediction is compromised. Since the influence of kerMAR is restricted to the corrupted regions, this cannot occur far from the dental implants, but it may occur in the trachea where the CT and MRI can be highly dissimilar due to anatomical variations between the scans (see yellow arrow in Fig. 2).

kerMAR is often robust to such errors as it includes a likelihood model of the corrupted CT values in addition to an MR‐based prior, and for each patient optimizes the relative weights of the two parts (see supplementary material, section 1). The errors occur when neither the prior nor likelihood are precise and accurate, that is, in regions that are simultaneously severely corrupted and poorly co‐registered. To overcome such issues, deformable image registration may substantially improve on the rigid mutual information co‐registration that we used in this study. Further improvements may include increasing patch size or improving the likelihood by, for example, better modeling the spatial variation in the noise over the image.

### 5.5 Future work

**Figure 8:**
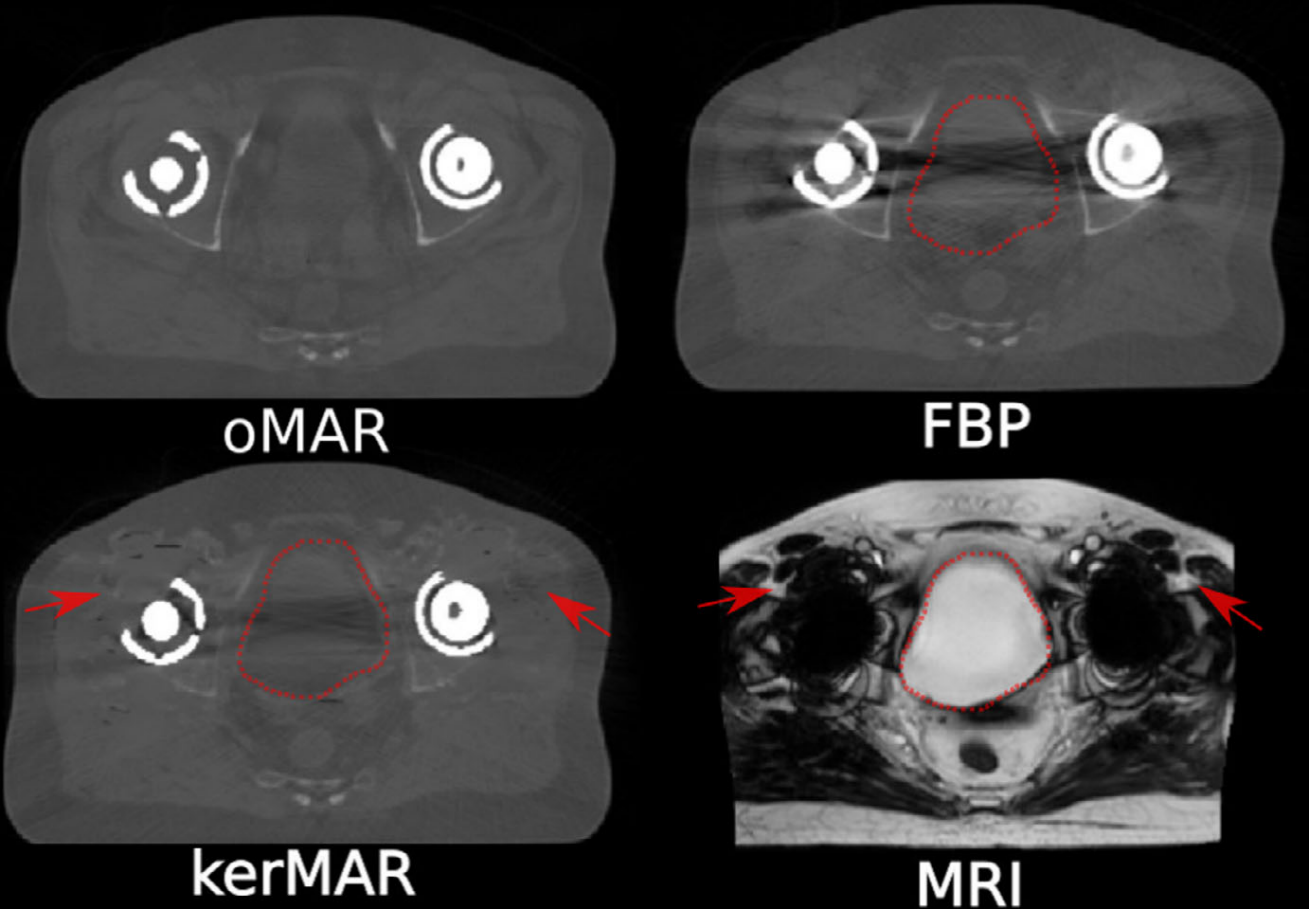
The Philips metal artifact reduction for orthopedic implants (oMAR), the filtered back projection (FBP), our magnetic resonance (MR)‐based kernel regression metal artifact reduction (kerMAR) and the T1w MR image (MRI) of a pelvis with metallic dual hip implants. The delineation of the bladder was performed in the MR image. The arrows point to regions where metal artifacts on the MRI led to artifacts in the kerMAR. [Color figure can be viewed at http://www.wileyonlinelibrary.com/]

We optimized kerMAR for the H&N RT application, which was therefore also the focus of this paper, leaving challenging cases such as (dual) hip implants as an interesting subject for further study. We show a preliminary result in Fig. 8, which highlights some of the challenges faced by the algorithm. First, the bladder, here delineated in the MRI, moved between acquisitions, leading to potentially erroneous soft tissue contrast in the kerMAR over a large region; second, the metal artifacts in the MRI are more extensive, leading to errors in the kerMAR (arrows); and third, the streaks are not well suppressed between the implants, which may be attributed to flaws in the modeled spatial variation of the artifact noise. Until these issues are properly addressed, kerMAR will likely not be as successful in the pelvis as we have seen in the H&N; the future work may therefore focus on these three potential improvements.

## 6 Conclusions

Based on phantom and head‐and‐neck patients, we investigated the impact of three different MAR strategies in terms of image quality and dosimetric impact for photon, electron and proton beams. We investigated the following three hypotheses: Hypothesis I, that is, “Water override is superior to the alternatives,” was supported for image quality in the soft tissue. However, the water override also led to phantom and patient depth/range errors of 1–3 mm.

Hypothesis II, “The automatic MARs oMAR and kerMAR were superior to the FBP,” was supported in terms of image quality in the soft tissue and patient teeth, accompanied by a significant impact on patient particle ranges of 1–1.5 mm. Hypothesis III, “kerMAR is superior to oMAR”, was supported for high intensity artifact reduction and accompanied by impacts on the electron and proton range estimates of 1–1.5 mm.

## Conflict of Interest

The authors have no relevant conflict of interest to disclose.

## Supporting information


**Fig. S1.** Schematic illustrations of (left–right) our magnetic resonance‐based kernel regression metal artifact reduction (kerMAR) algorithm, the computed tomography‐based Philips metal artifact reduction for orthopedic implants (oMAR) algorithm and manual water override.

**Fig. S2.** Muscle override at 60 HU results on the veal shank phantom. (a) Image analysis. (b) Dose calculations. (c) An axial slice using muscle override. (d) The corresponding slice with water override.

**Fig. S3.** Evaluation of our cubic interpolation strategy to detect subresolution differences between depth‐dose curves. (a): Cubically interpolated proton depth‐dose curves (Bragg peaks), for metal artifact reduction for orthopedic implants (oMAR) and kernel regression metal artifact reduction (kerMAR) and three patients. The resolution of the data points is 2 mm (closed circles). (b): Simulation of a lambda‐distribution (approximating a Bragg peak) with data points down‐sampled to a 2mm resolution and cubically interpolated.
