# Supplementary figures and images for: MR‐based CT metal artifact reduction for head‐and‐neck photon, electron, and proton radiotherapy
Source: Med Phys. 2019 Aug 10;46(10):4314–23. doi: 10.1002/mp.13729 (PMC6802740; doi:10.1002/mp.13729)

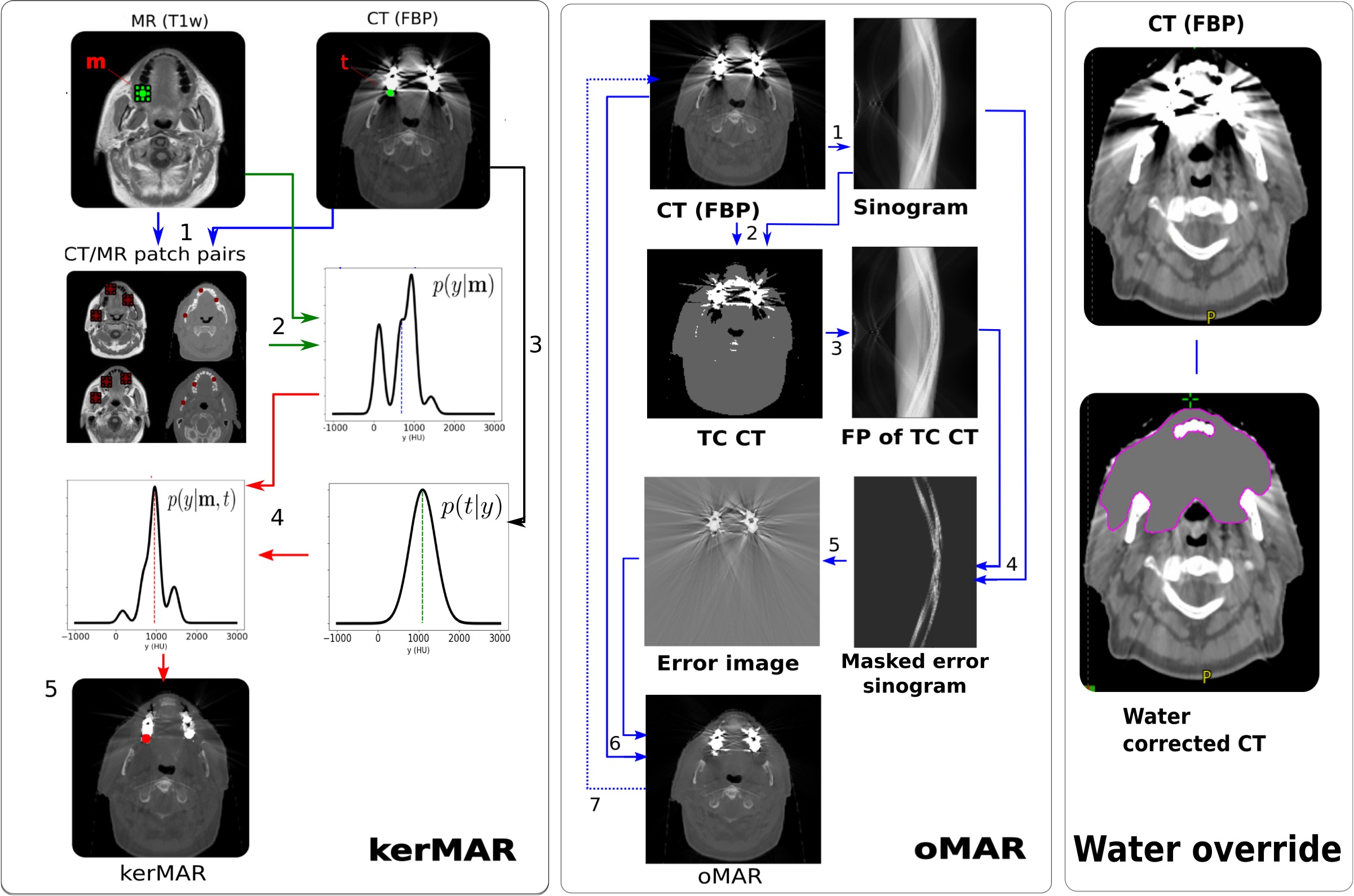

Supplement: Supplementary file 1 — Fig. S1. Schematic illustrations of (left–right) our magnetic resonance‐based kernel regression metal artifact reduction (kerMAR) algorithm, the computed tomography‐based Philips metal artifact reduction for orthopedic implants (oMAR) algorithm and manual water override. [file MP-46-4314-s001.jpg]

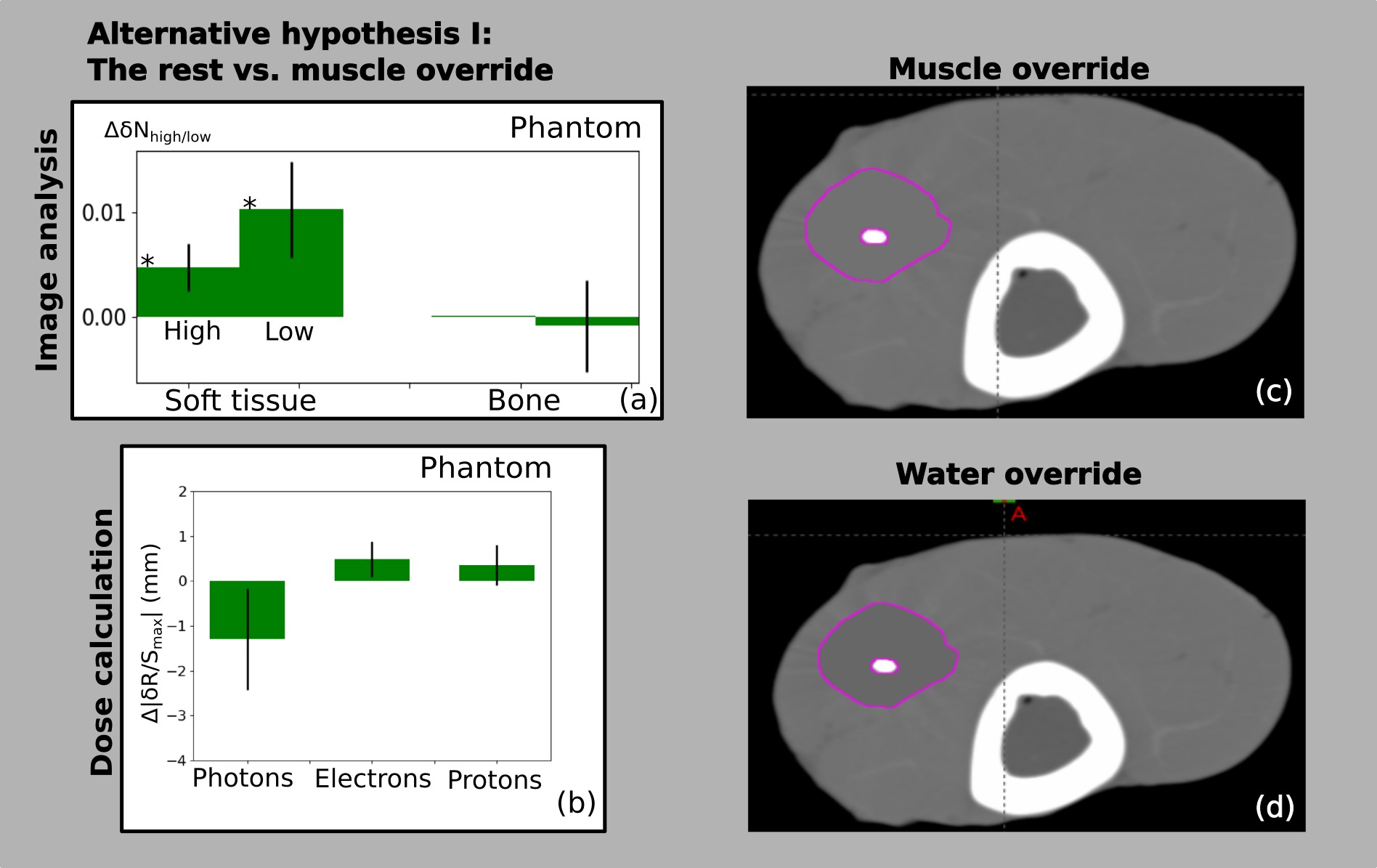

Supplement: Supplementary file 2 — Fig. S2. Muscle override at 60 HU results on the veal shank phantom. (a) Image analysis. (b) Dose calculations. (c) An axial slice using muscle override. (d) The corresponding slice with water override. [file MP-46-4314-s002.jpg]

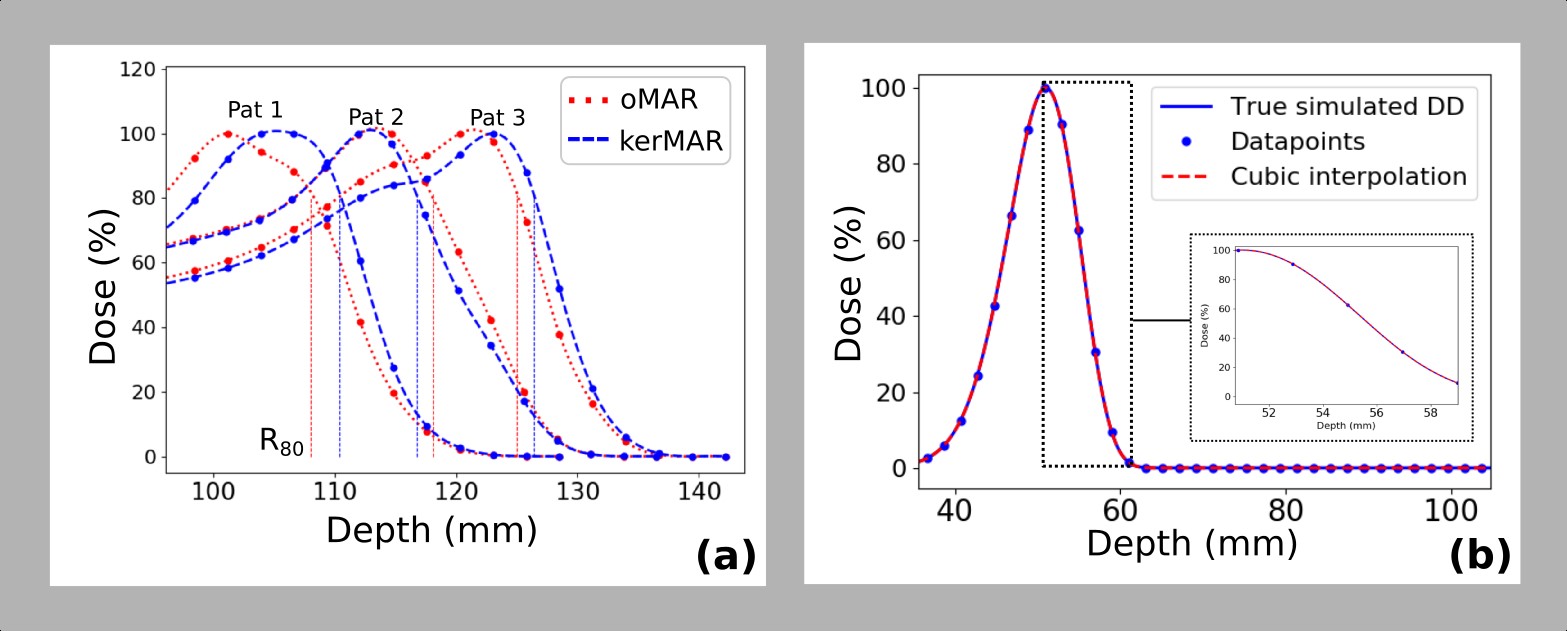

Supplement: Supplementary file 3 — Fig. S3. Evaluation of our cubic interpolation strategy to detect subresolution differences between depth‐dose curves. (a): Cubically interpolated proton depth‐dose curves (Bragg peaks), for metal artifact reduction for orthopedic implants (oMAR) and kernel regression metal artifact reduction (kerMAR) and three patients. The resolution of the data points is 2 mm (closed circles). (b): Simulation of a lambda‐distribution (approximating a Bragg peak) with data points down‐sampled to a 2mm resolution and cubically interpolated. [file MP-46-4314-s003.jpg]
