# Supplementary material for: MR‐based CT metal artifact reduction for head‐and‐neck photon, electron, and proton radiotherapy
Source: Med Phys. 2019 Aug 10;46(10):4314–23. doi: 10.1002/mp.13729 (PMC6802740; doi:10.1002/mp.13729)
Supplement: Supplementary file 4 [file MP-46-4314-s004.doc]

Supplementary material

1. The metal artifact reduction algorithms

Here, we provide an overview of the three metal artifact reduction (MAR) approaches considered in this study.

1. **Kernel regression MAR**

The Magnetic Resonance (MR)-based kernel regression MAR (kerMAR) ^1^ consists of the following steps (see Fig. 1):

1. **Patch matching:** The axial slices that contain metal implants are identified by thresholding the FBP. Then, for each voxel in these slices (example marked by *t*), a set of CT value/MRI patch regression point pairs are found in the nearby, uncorrupted volume.
2. **Kernel density estimation and kernel regression:** From the set of regression points, the joint distribution *p(y,* ***m****)* is estimated by kernel density estimation (KDE) with Gaussian kernels^3^. Given this distribution and an observed MRI patch **m** at the corrupted voxel location, the prior distribution of the clean CT values *y*, *p(y|****m****)*, may be found; this is known as *kernel regression^3^.* The expectation value of y under this model is indicated with a blue vertical dotted line.
3. **Noise modelling:** The corrupted CT measurement *t* is assumed to arise from the unknown, true CT value *y* from a Gaussian noise model *p(t|y)*. The variance of this noise model is set to decrease with the distance to the metal implants from a maximal level near the artifacts, such that it vanishes for voxels in uncorrupted, far-lying regions (expectation value indicated with green vertical dotted line).
4. **Posterior modelling:** The posterior distribution *p(y|t,****m****)* is constructed for each voxel from the prior *p(y|****m****)* and *p(t|y)* via Bayes formula.
5. **Bayesian estimation:** The expectation value of *y* is calculated over the posterior to yield the replacement CT values (indicated with red vertical dotted line).

**Implementation and automatic hyperparameter settings:**

Steps 2-5 occur in practice by evaluating an analytical expression for *y* given the imaging data and the settings of three critical *hyperparameters* of the model. These are in particular 1) the variances of the CT and MRI kernels in the KDE, which effectively control the width and placement of the peaks in *p(y|****m****)***,** and 2) the maximal artifact noise variance that affects the width of *p(t|y)*. They are automatically set for each patient using Empirical Bayes^4,5^ estimation, which maximizes their probability given the data. This tunes the model to each individual patient and takes into account e.g. the artifact corruption level and systematic image contrast variations due to the choice of the MR sequence.


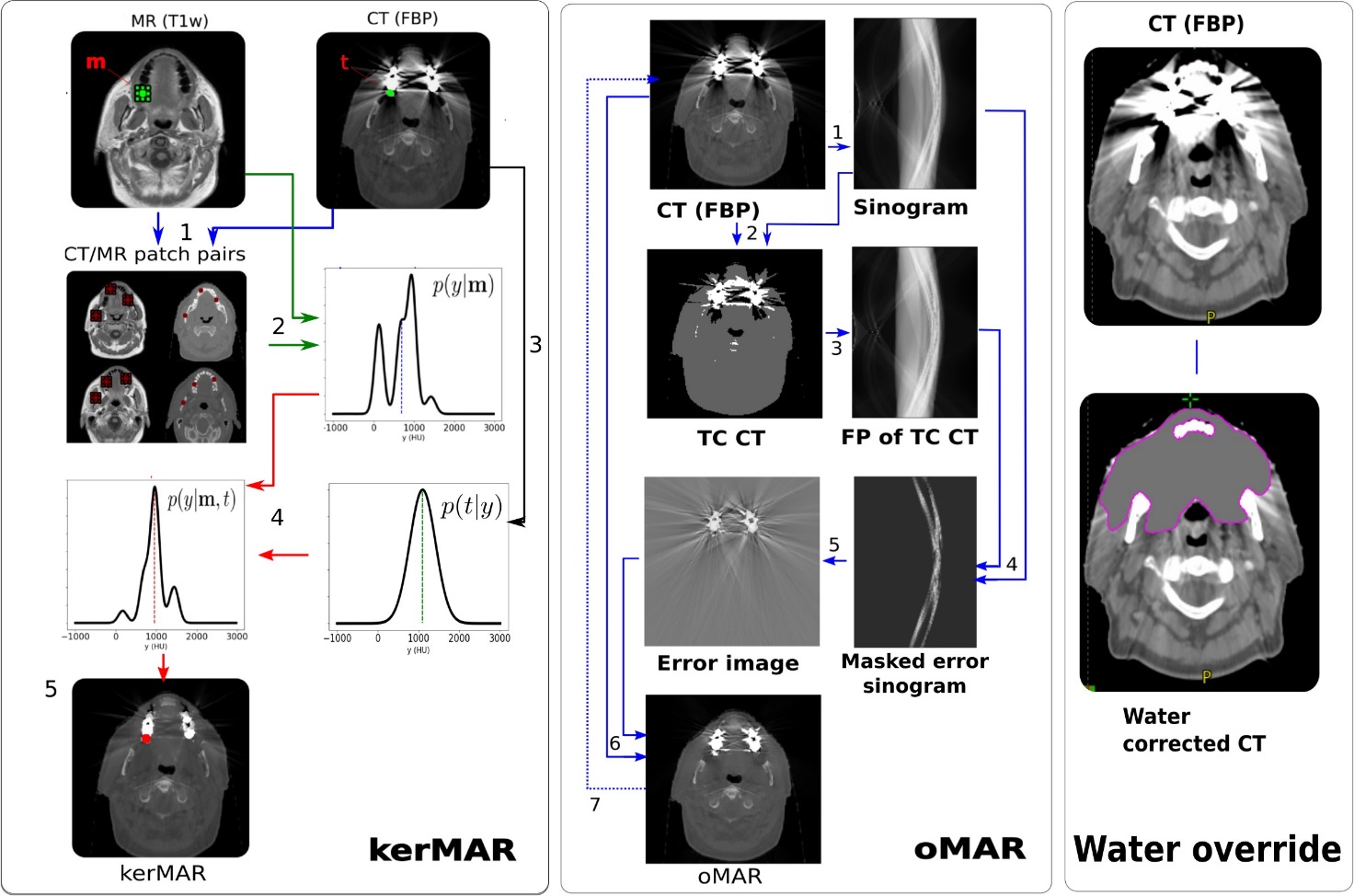


FIG. S-1. Schematic illustrations of (left-right) our MR-based kernel regression metal artifact (kerMAR algorithm), the CT-based Philips metal artifact reduction for orthopedic implants (oMAR) algorithm and manual water override.

1. **MAR for orthopedic implants**

MAR for orthopedic implants (oMAR, Philips Healthcare) is an iterative algorithm^2^ that proceeds as follows:

1.) **Forward project** (FP) through the input CT image (reconstructed by FBP) to simulate the sinogram.

2.) **Segment** the input CT to get a tissue classified prior image (TC CT). This step includes in practice a preliminary artifact reduction using a sinogram inpainting method that improves the segmentation.

3.) **FP** through the prior to yield a prior sinogram.

4.) **Subtract** **the prior sinogram** from the original sinogram to create an error sinogram. The error sinogram is here also masked by an FP through a metal segmentation to localize the artifact reduction to artifact-affected regions.

5.) **Reconstruct the** **error image** from the error sinogram.

6.) **Subtract the error image** from the FBP.

7.) **Replace the input CT** with the updated image for iteration, or stop here.

1. **Water override**

Water override proceeds as follows: replace any visibly corrupted soft tissue regions, as well as any intensely corrupted regions, with 0HU.

1. Muscle override in the phantom

In our evaluation of the manual approach to MAR, we performed water override with 0HU. Since the veal shank phantom primarily contained muscle, this led to a systematic error of approximately 60HU, as is evident when comparing figs. 2c and 2d where we respectively used muscle override with 60HU and water override with 0HU.

We accordingly tested hypothesis I (manual override was superior to the competing techniques) using the muscle override. The image quality results in fig. 2a are identical to the water override results, which is as expected since the change in override values does not affect the outliers of the HU value distributions that are captured by the image quality metrics. The dose results in fig. 2b do, however, improve over the water override shown in fig. 5b of the manuscript, especially for electrons and protons. The improvement however remains insufficient to support the hypothesis.


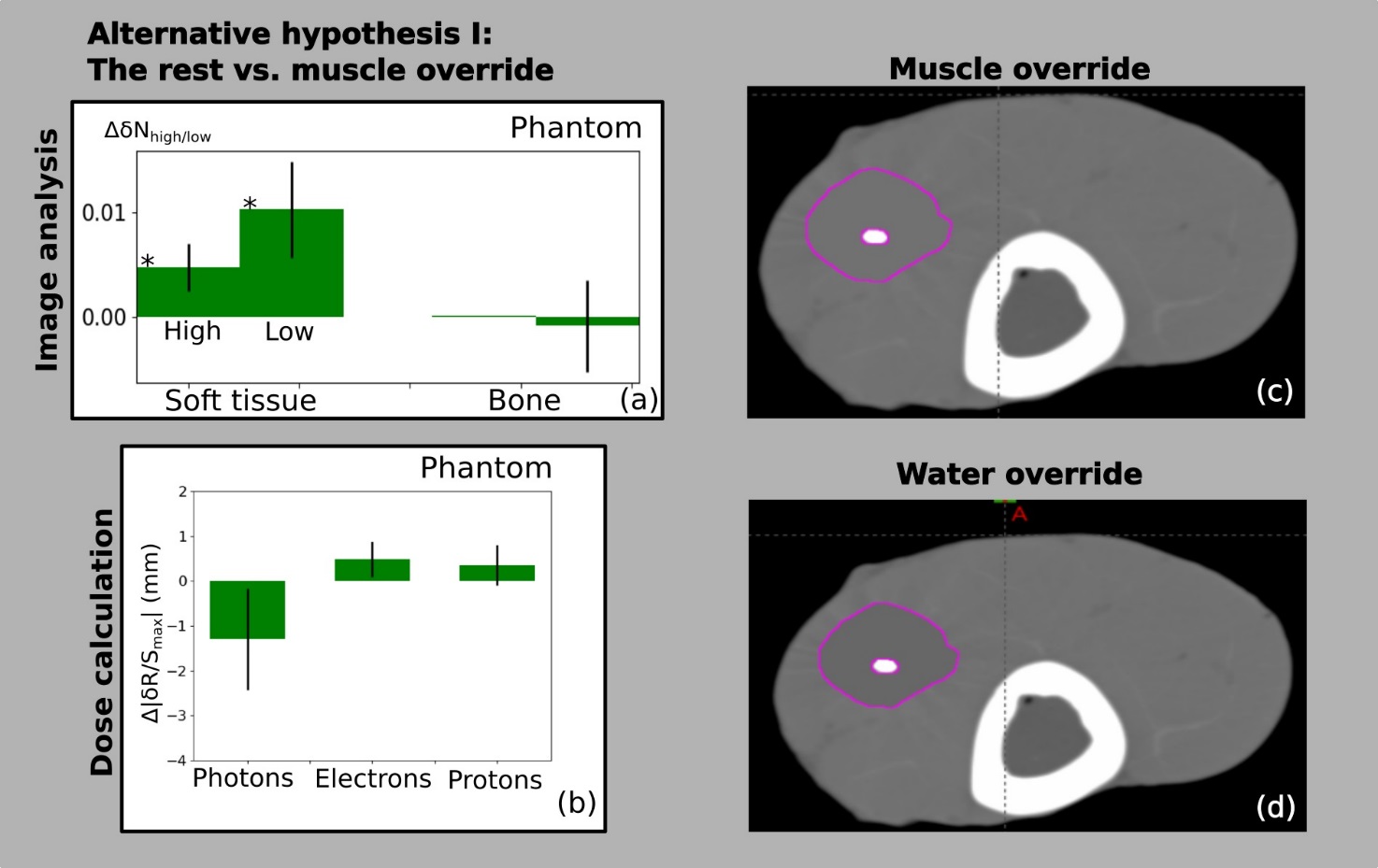


FIG S-2. Muscle override at 60HU results on the veal shank phantom. (a): Image analysis. (b): Dose calculations. (c): An axial slice using muscle override. (d): The corresponding slice with water override.

1. evaluation of the depth-dose curve interpolation

A concern when calculating the photon depth and particle ranges in our experiments was the relatively low spatial resolution of the depth-dose curves (2mm). Since the deviations were typically below this resolution, we calculated the depths and ranges on cubically interpolated versions of the depth-dose curves. We show a few examples of this in fig. 3a for the proton Bragg peaks which were also the sharpest varying of the depth-dose-curves and thus the most error prone.

The cubic interpolation here appears to be a good fit to the data points, and it is possible to distinguish the oMAR and kerMAR curves and calculate sub-resolution deviations.

There may however be errors in the cubic interpolation. To investigate this, we simulated a Bragg peak-like lambda-distribution with similarly spaced, down-sampled data points as in the experiments (2mm resolution), and the same cubic interpolation (fig. 3b). The errors of the cubic interpolation are here negligible, which implies that the interpolation errors are minor.


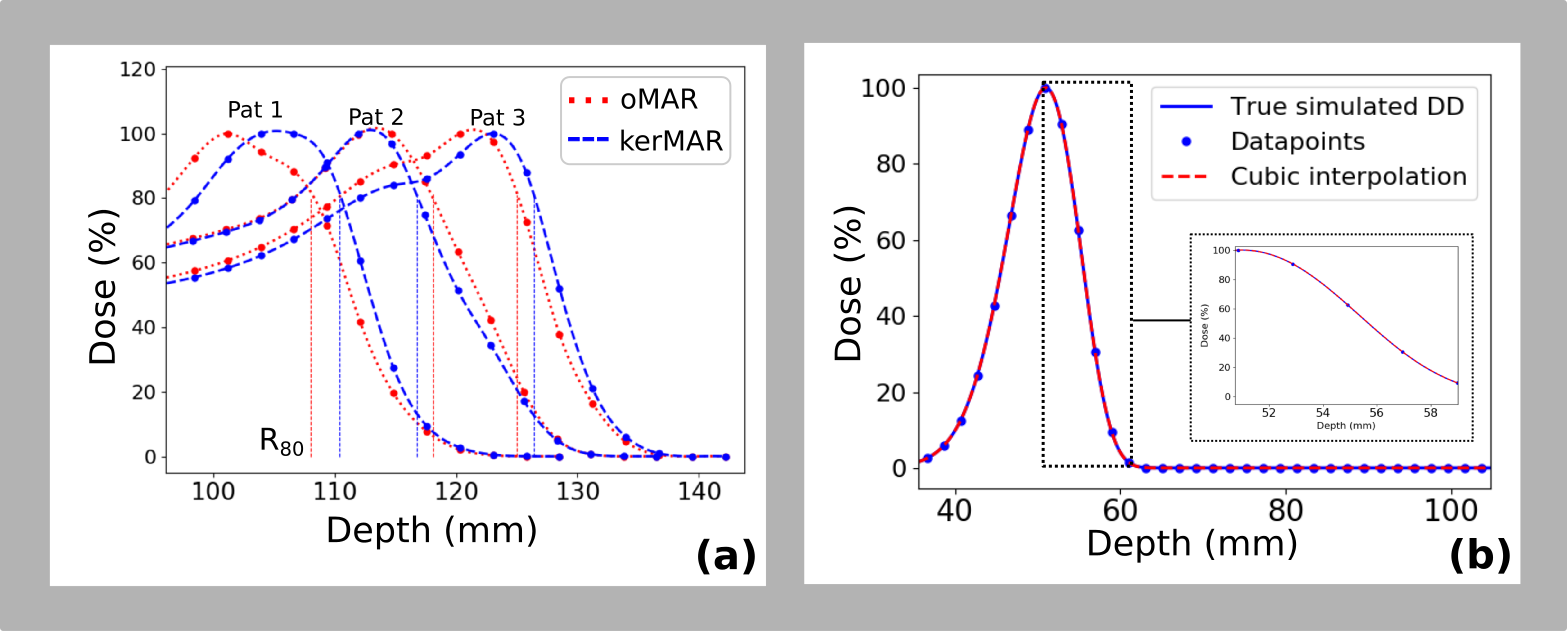


FIG S-3. Evaluation of our cubic interpolation strategy to detect sub-resolution differences between depth-dose-curves. (a): Cubically interpolated proton depth-dose curves (Bragg peaks), for metal artifact reduction for orthopedic implants (oMAR) and kernel regression metal artifact reduction (kerMAR) and three patients. The resolution of the data points is 2mm (closed circles). (b): Simulation of a lambda-distribution (approximating a Bragg peak) with data points down-sampled to a 2mm resolution and cubically interpolated.

1. References
2. Nielsen J, Edmund J, Van Leemput K. *CT metal artifact reduction using MR image patches*, vol. 10573. SPIE - International Society for Optical Engineering, 2018[; doi:10.1117/12.2293815.](http://dx.doi.org/10.1117/12.2293815)
3. Philips Healthcare. *Metal artifact reduction for orthopedic implants (omar)*. [http:](http://clinical.netforum.healthcare.philips.com/us_en/Explore/ White-Papers/CT/Metal-Artifact-Reduction-for-Orthopedic-Implants-(O-MAR))

[//clinical.netforum.healthcare.philips.com/us_en/Explore/White-Papers/CT/](http://clinical.netforum.healthcare.philips.com/us_en/Explore/ White-Papers/CT/Metal-Artifact-Reduction-for-Orthopedic-Implants-(O-MAR)). [Metal-Artifact-Reduction-for-Orthopedic-Implants-(O-MAR),](http://clinical.netforum.healthcare.philips.com/us_en/Explore/ White-Papers/CT/Metal-Artifact-Reduction-for-Orthopedic-Implants-(O-MAR)) 2012. Accessed August 2018.

1. Bishop CM. Pattern recognition and machine learning. Springer, 2016; ISBN 0387310738, 9780387310732.
2. Robert, CP. The Bayesian Choice. A decision-theoretic motivation. Springer-Verlag, 1994; ISBN 0387842963, 3540942963
3. MacKay, JC. Information Theory, Inference and Learning Algorithms, Volume 100, 2005; ISBN 9780521642989; URL
   http://pubs.amstat.org/doi/abs/10.1198/jasa.2005.s54{%}5Cnhttp://www.cambridge.org/0521642981
